# Impact of dose reductions on adjuvant abemaciclib efficacy for patients with high-risk early breast cancer: analyses from the monarchE study

**DOI:** 10.1038/s41523-024-00639-1

**Published:** 2024-04-26

**Authors:** Matthew P. Goetz, Irfan Cicin, Laura Testa, Sara M. Tolaney, Jens Huober, Valentina Guarneri, Stephen R. D. Johnston, Miguel Martin, Priya Rastogi, Nadia Harbeck, Ashwin Shahir, Ran Wei, Valérie André, Hope S. Rugo, Joyce O’Shaughnessy

**Affiliations:** 1 Department of Oncology, Mayo Clinic, Rochester, MN USA; 2 Istinye University Faculty of Medicine, Istanbul, Turkey; 3 Instituto D’Or de Pesquisa e Ensino (IDOR), São Paulo, Brazil; 4 Dana-Farber Cancer Institute, Boston, MA USA; 5 Cantonal Hospital St. Gallen, Breast Centre St. Gallen, St. Gallen, Switzerland; 6 Department of Gynecology and Obstetrics, University of Ulm, Ulm, Germany; 7 Department of Surgery, Oncology and Gastroenterology, University of Padova, Padova, Italy; 8 Istituto Oncologico Veneto IRCCS, Padova, Italy; 9 Royal Marsden NHS Foundation Trust, London, United Kingdom; 10 Hospital General Universitario Gregorio Marañon, Universidad Complutense, CIBERONC, GEICAM, Madrid, Spain; 11 University of Pittsburgh/UPMC, NSABP Foundation, Pittsburgh, PA USA; 12 Breast Centre, Department of Gynaecology and Obstetrics, Comprehensive Cancer Centre München, LMU University Hospital, Munich, Germany; 13 Loxo@Lilly, Indianapolis, IN USA; 14 Eli Lilly and Company, Indianapolis, IN USA; 15 University of California San Francisco Hellen Diller Family Comprehensive Cancer Center, San Francisco, CA USA; 16 Baylor University Medical Center, Texas Oncology, US Oncology, Dallas, TX USA

**Keywords:** Breast cancer, Targeted therapies

## Abstract

In monarchE, adjuvant abemaciclib significantly improved invasive disease-free survival (IDFS) and distant relapse-free survival (DRFS), with sustained benefit beyond the 2-year treatment period. Abemaciclib dose reductions were allowed to proactively manage adverse events. Exploratory analyses to investigate the impact of dose reductions on efficacy were conducted. Across the three patient subgroups as defined by relative dose intensity (≤66%, 66–93%, ≥93%), the estimated 4-year IDFS rates were generally consistent (87.1%, 86.4%, and 83.7%, respectively). In the time-dependent Cox proportional hazard model, the effect of abemaciclib was consistent at the full dose compared to being reduced to a lower dose (IDFS hazard ratio: 0.905; 95% confidence interval: 0.727, 1.125; DRFS hazard ratio: 0.942; 95% confidence interval: 0.742, 1.195). These analyses showed that the efficacy of adjuvant abemaciclib was not compromised by protocol mandated dose reductions for patients with node positive, hormone receptor positive, human epidermal growth factor 2-negative, high-risk early breast cancer.

## Introduction

The addition of 2 years of abemaciclib to standard adjuvant endocrine therapy (ET) for high-risk, hormone receptor-positive (HR+), human epidermal growth factor receptor negative (HER2-) early-stage breast cancer (EBC) resulted in significant improvements in invasive disease-free survival (IDFS) and distant relapse-free survival (DRFS), which were further strengthened after the 2-year treatment period^1,2^. Notably, at a median follow-up of 42 months, improvements in IDFS and DRFS represented a relative risk reduction of 34% for disease recurrence or distant metastases in the abemaciclib plus ET arm compared to the ET alone arm^2^. Based on these results, abemaciclib is currently the only cyclin-dependent kinase 4/6 dual inhibitor approved as adjuvant therapy for node-positive, HR+, HER2-, high-risk EBC^3–5^, with a National Comprehensive Cancer Network Category 1 rating^6^ and a maximum score (A) from the European Society for Medical Oncology on the Magnitude of Clinical Benefit Scale^7^.

The goal of adjuvant treatment is to eliminate micro-metastatic disease to prevent recurrence; thus, the failure to retain patients on abemaciclib for the full 2-year treatment period could compromise treatment efficacy. It has also been established previously, that abemaciclib dose modifications improve tolerability without negatively affecting progression-free survival (PFS) in the metastatic breast cancer setting^8^.

Similar to the safety profile of abemaciclib plus ET in the advanced and metastatic breast cancer setting^8^, most adverse events in the EBC setting were reversible and manageable with supportive medications and/or dose modifications^9^. In monarchE, dose reductions due to adverse events occurred in 43.4% of patients treated with abemaciclib, most frequently in response to diarrhea, neutropenia, and/or fatigue^9^. Although dose reductions occurred at different timepoints, the majority took place within the first 6 months^9^. Abemaciclib dose reductions were shown to effectively manage adverse events, with only a small proportion of patients discontinuing after a dose reduction (8.9%), indicating that early dose reductions may improve treatment adherence^9^. In contrast, 52% of patients who discontinued abemaciclib due to an adverse event did not have a prior dose reduction, including 88% of patients who discontinued during the first month of treatment^9^.

However, the patient disease characteristics associated with dose reductions, as well as the impact of abemaciclib dose reductions on efficacy during adjuvant EBC treatment have not been previously described. In this post hoc analysis we evaluated baseline patient disease characteristics to identify patients who could benefit from more frequent monitoring of side effects and, if required, earlier dose reductions. More importantly, we report exploratory analyses to determine the impact of dose modifications, specifically dose reductions, on the efficacy of adjuvant abemaciclib for the treatment of high-risk, node-positive, HR+, HER2- EBC.

## Results

### Patient characteristics by the number of dose reductions

In the monarchE trial, 2791 patients were treated with adjuvant abemaciclib plus ET. Of these, 1221 (43.7%) had dose reductions, including 832 (29.8%) with one and 389 (13.9%) with two dose reductions. Dose reductions were most commonly required for diarrhea (17.3%), neutropenia (8.1%), or fatigue (4.5%)^9^.

Table 1 shows patient demographics and clinical characteristics by the number of dose reductions. For all reported demographics and clinical characteristics except age and pre-existing comorbidities, distributions of characteristics were similar across the dose reduction groups. Higher proportions of patients ≥65 years old or with ≥4 pre-existing comorbidities were observed among patients with dose reductions. In addition, the likelihood of dose reductions within each patient characteristic factor was assessed: 55.8% of older patients and 49.9% of patients with ≥4 comorbidities had at least one dose reductions.

**Table 1:** Patient demographics and clinical characteristics by number of abemaciclib dose reductions

Characteristics	No dose reduction *N* = 1570	One dose reduction *N* = 832	Two dose reductions *N* = 389
**Age group**			
<65 years old	1380 (87.9)	683 (82.1)	298 (76.6)
≥65 years old	190 (12.1)	149 (17.9)	91 (23.4)
**Prior chemotherapy**			
Neoadjuvant chemotherapy	580 (36.9)	315 (37.9)	137 (35.2)
Adjuvant chemotherapy	922 (58.7)	483 (58.1)	228 (58.6)
No chemotherapy	68 (4.3)	34 (4.1)	24 (6.2)
**Pre-existing comorbidities**			
None	294 (18.7)	117 (14.1)	49 (12.6)
1–3 comorbidities	796 (50.7)	396 (47.6)	181 (46.5)
≥4 comorbidities	480 (30.6)	319 (38.3)	159 (40.9)
**Number of positive nodes**^**a**^			
1–3 nodes	616 (39.2)	323 (38.8)	176 (45.2)
≥4 nodes	949 (60.4)	507 (60.9)	213 (54.8)
**Pathological tumor size**^**b**^			
<20 mm	444 (28.3)	221 (26.6)	110 (28.3)
≥20 mm	1094 (69.7)	599 (72.0)	275 (70.7)
**Histological tumor grade**^**c**^			
Grade 1	126 (8.0)	50 (6.0)	33 (8.5)
Grade 2	791 (50.4)	413 (49.6)	167 (42.9)
Grade 3	580 (36.9)	329 (39.5)	166 (42.7)

Data cutoff date: July 01, 2022 N, number of patients; n, number of patients in the group ^a^Patients with no nodes were not included in this table, ^b^Patients with missing tumor size information were not included in this table, ^c^Patients with missing on non-assessable tumor grade were not included in this table.

### Abemaciclib exposure by the number of dose reductions

The median duration of abemaciclib treatment was 23.7 months, regardless of dose reductions. Compared to those with no dose reduction, a greater proportion of patients with one or two reductions completed the first 6 months of treatment (Table 2). At later time points, treatment retention was comparable, or even improved, in patients with dose reductions (Table 2).

**Table 2:** Abemaciclib exposure by the number of dose reductions

	No dose reduction *N* = 1570	One dose reduction *N* = 832	Two dose reductions *N* = 389
**Treatment duration, months**			
Median	23.7	23.7	23.7
Q1–Q3	14.9–23.8	20.6–23.8	13.2–23.8
>3 months, *n* (%)	1349 (85.9)	787 (94.6)	367 (94.3)
>6 months, *n* (%)	1276 (81.3)	750 (90.1)	333 (85.6)
>12 months, *n* (%)	1200 (76.4)	677 (81.4)	297 (76.3)
>18 months, *n* (%)	1146 (73.0)	637 (76.6)	274 (70.4)
**Cumulative dose, mg**			
Median	192,450	137,475	77,200
Q1–Q3	112,900–210,900	98,825–151,950	50,100–96,500
**Relative dose intensity**^**a**^**, %**			
Median	94.6	66.5	40.2
Q1–Q3	83.4–99.0	59.5–74.4	34.5–50.7

Data cutoff date: July 01, 2022.

N, number of patients; n, number of patients in the group; Q1–Q3, quartile 1 to quartile 3 range ^a^Relative dose intensity was defined as the average daily dose of abemaciclib received by each patient over the treatment duration, relative to the full dose (150 mg twice per day). Dose reductions of up to two 50-mg dose levels (100 or 50 mg) were permitted during the on-study treatment period.

Patients with dose reductions had a lower cumulative dose and RDI compared to those without (median RDI: 94.6%, 66.5%, and 40.2% in the no, one, and two dose reduction subgroups, respectively; Table 2).

### Efficacy by patient subgroups defined by relative dose intensity

**Fig. 1:**
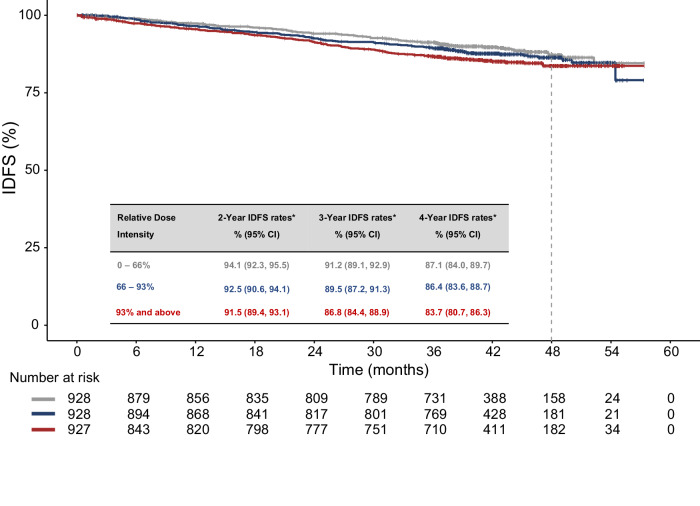
Invasive disease-free survival by relative dose intensity subgroup in patients treated with abemaciclib. RDI was defined as the average daily dose of abemaciclib received by each patient over the treatment duration, relative to the full dose (150 mg twice per day). Among the 2791 abemaciclib-treated patients, 2783 had complete treatment exposure information for RDI calculation and thus were included in this analysis. *Estimated by the Kaplan-Meier method. For efficacy analyses, patients were divided into three equal-sized subgroups according to their abemaciclib RDI. Data cutoff date: July 01, 2022. CI confidence interval, IDFS invasive disease-free survival, RDI relative dose intensity.

According to the Kaplan-Meier plots of IDFS by RDI subgroups (Fig. 1), the effect of abemaciclib was generally consistent across RDI subgroups with no clinically meaningful differences in the estimated 4-year IDFS rates between the RDI subgroups (4-year rates [95% CI]: 87.1% [84.0%, 89.7%], 86.4% [83,6%, 88.7%], and 83.7% [80.7%, 86.3%], respectively; Supplementary Table 1). Similar findings were observed in abemaciclib-treated patients in Cohort 1 (Supplementary Table 1 and Supplementary Figure 2).

### Impact of dose reductions on efficacy using a time-dependent model

According to the time-dependent Cox proportional hazards model that included dose levels with their start and end time as the only variable, the abemaciclib benefit was consistent at the 150 mg full dose, compared to the reduced doses of 100 mg or 50 mg (unadjusted hazard ratio [95% CI] IDFS: 0.905 [0.727, 1.125]; DRFS: 0.942 [0.742, 1.195]; Table 3). These results were further supported by a time-dependent Cox PH model adjusted by baseline age, stratification factors, key disease characteristics, and pre-existing co-morbidities (Table 3). Similar findings were observed in Cohort 1 patients for both adjusted and unadjusted estimates (Table 3).

**Table 3:** Time-dependent Cox PH model for the impact of dose reductions on Invasive disease-free survival and distant relapse-free survival

Efficacy Endpoint	Assessment of Efficacy Staying at full dose vs Being reduced to a lower dose	
	Unadjusted Hazard Ratio (95% CI)^a^	Adjusted Hazard Ratio (95% CI)^b^
**Patients treated with abemaciclib in Intent-to-treat population**		
IDFS	0.905 (0.727, 1.125)	0.922 (0.740, 1.148)
DRFS	0.942 (0.742, 1.195)	0.954 (0.751, 1.212)
**Patients treated with abemaciclib in Cohort 1**^**c**^		
IDFS	0.899 (0.718, 1.125)	0.918 (0.732, 1.150)
DRFS	0.958 (0.750, 1.223)	0.972 (0.76, 1.243)

Data cutoff date: July 01, 2022.

*ALN* axillary lymph node, *CI* confidence interval, *DRFS* distant relapse-free survival, *IDFS* invasive disease-free survival.

^a^Hazard ratio (95% CI) was estimated using a time-dependent Cox proportional hazards model to assess the impact of dose levels over time on IDFS and DRFS.

^b^Adjusted by confounding baseline factors individually associated with risk of recurrence, including age, stratification factors, key disease characteristics, and pre-existing co-morbidities.

^c^Cohort 1 included patients with ≥4 positive pathologic ALNs or 1-3 positive ALNs plus tumor size ≥5 cm and/or tumor grade 3.

In monarchE, 25.8% of patients discontinued abemaciclib due to reasons other than recurrence, including 18.5% due to AEs^9^. Additionally, the multivariate analysis of TTD identified that age ≥65 years, postmenopausal status, ≥4 pre-existing comorbidities, enrolled in North America or Europe, baseline Eastern Cooperative Oncology Group performance status (ECOG PS) of 1, and presence 1–3 positive ALN were independently associated with greater risk of abemaciclib discontinuation (Table 4). Older age was associated with the greatest increase in risk of treatment discontinuation. For the selected factors, discontinuation rates between subgroups within each factor diverged early and continued to separate during the 2-year treatment period with the highest rates of early discontinuation occurring in those aged ≥65 years old and/or with ≥4 pre-existing comorbidities (Supplementary Table 2**;** Supplementary Figure 3).

**Table 4:** Multivariate analysis of factors associated with the risk of discontinuation in abemaciclib-treated patients

Factors		Hazard ratio (95% CI)	Multivariate Model^a,b^ *P* value
Geographic region	Asia vs NA/Europe	0.671 (0.541, 0.834)	<0.0001
	Other vs NA/Europe	0.672 (0.557, 0.811)	
Menopausal status	Post- vs premenopausal	1.514 (1.268, 1.806)	<0.0001
Age group	≥65 years vs <65 years	1.879 (1.566, 2.256)	<0.0001
Baseline ECOG PS	0 vs 1	0.801 (0.662, 0.971)	0.0236
Number of positive nodes	4–9 vs 1–3	0.806 (0.685, 0.949)	<0.0001
	≥10 vs 1–3	0.635 (0.514, 0.784)	
Number of unique pre-existing comorbidities	1–3 vs 0	1.213 (0.940, 1.566)	0.0004
	≥4 vs 0	1.563 (1.203, 2.032)	

Data cutoff date: July 01, 2022.

*CI* confidence interval, *ECOG PS* Eastern Cooperative Oncology Group performance status, *NA* North America; vs, versus.

^a^Included factors with *P* value < 0.05 in univariate analyses, selected in a stepwise fashion based on a multivariate Cox model, with an entry and retaining *P* value threshold of 0.05.

^b^Wald’s *P* value.

To further explore the impact of dose reductions on efficacy in the adjuvant setting, any IDFS events occurring beyond the abemaciclib treatment period were censored at the time of abemaciclib completion as a sensitivity analysis. In a time-dependent Cox proportional hazards model adjusted by the factors associated with an increased discontinuation, the effect of abemaciclib during the 2-year treatment period was consistent at 150 mg, compared to the reduced doses of 100 mg or 50 mg (hazard ratio [95% CI] IDFS: 0.821 [0.597, 1.129]; DRFS: 0.804 [0.564, 1.145]).

## Discussion

In the monarchE trial, adjuvant abemaciclib plus ET significantly improved IDFS and DRFS compared to adjuvant ET alone in patients with high-risk, HR+, HER2- EBC with sustained benefit beyond the 2-year abemaciclib treatment. The well-established safety profile of adjuvant abemaciclib is considered predictable, manageable, and acceptable in the high-risk EBC patient population^8^. Here, we provide comprehensive analyses assessing the impact of abemaciclib dose modifications, which are essential in the management of toxicities to maximize treatment adherence and retain patients on treatment to achieve optimal benefit. Notably, consistent with the findings in the metastatic setting^8^, our analyses suggest that the efficacy of adjuvant abemaciclib in high-risk EBC is not compromised by dose reductions.

Since dose reductions are common, it is critical to understand any potential impact of receiving a reduced adjuvant abemaciclib dose on efficacy. This unanswered question presents multiple challenges. First, the timing, number, and duration of dose reductions vary between patients. Second, dose reductions and treatment durations are positively correlated as patients with dose reductions were likely to remain on treatment longer and patients remaining on treatment were more likely to have had a dose reduction. Thus, direct comparisons of efficacy between patients who did and did not have dose reductions could be biased and were not conducted here. To take into account the timing and duration of the dose reduction data, several statistical analyses were applied. In the first instance, given the decreasing trend of RDI by the number of dose reductions, an efficacy analysis by patient subgroups defined by RDI was considered an indirect way to assess the impact of dose modifications on efficacy. Next, to evaluate the relationship between efficacy and dose levels more directly, a time-dependent Cox proportional hazards model was implemented. This more complex analytical approach incorporated the start and end time of each dose level and assessed the effect of staying at full dose in comparison with being reduced to a lower dose. Furthermore, additional sensitivity analyses using the time-dependent Cox model were performed to adjust for confounding effects of baseline characteristics that were potentially associated with dose reductions.

Importantly, multiple analyses assessing the impact of dose reductions on efficacy reached the same conclusion, confirming that the benefit of abemaciclib is consistent whether at the full 150 mg dose or reduced to 100 mg or 50 mg. Of note, the efficacy analysis by RDI subgroups showed numerically higher 4-year IDFS rates among patients with lower RDI for abemaciclib. However, as the confidence intervals around the estimates overlap across these subgroups, there is no evidence suggesting different efficacy across RDI subgroups. These results provide evidence that the treatment benefit is not compromised by dose reductions (made in accordance with the protocol) and is consistent with previous observations in the metastatic breast cancer^8^.

These observations are clinically relevant as early discontinuation and non-adherence rates for adjuvant ET are high and often unrecognized^10,11^. Treatment adherence in the adjuvant ET setting has also been reported to be critical to ensure benefit^12^. In monarchE, approximately 25% of patients discontinued abemaciclib before completing 2-years of treatment for reasons other than tumor recurrence (18.5% due to AEs)^9^. Most discontinuations occurred early in the treatment period and usually in the first months. Various factors were identified as independently prognostic of discontinuation from the 2-year treatment period in abemaciclib-treated patients, including ≥4 pre-existing comorbidities, age ≥65 years, baseline ECOG PS of 1, and postmenopausal status. Patients with any of these features should be closely monitored for symptoms with early interventions. Instead of discontinuing patients from treatment due to toxicity, dose reductions should be considered to manage side effects and improve treatment adherence.

With the goal of improving tolerability and retaining patients on treatment, abemaciclib dose reductions were commonly implemented to manage side effects particularly in patients ≥65 years old or with ≥4 pre-existing comorbidities. We have previously reported that patients with dose reductions generally completed the 2-year abemaciclib treatment. Only 8.9% of patients discontinued due to adverse events following dose reduction^9^. Furthermore, two-thirds of abemaciclib discontinuations due to adverse events were in response to low-grade events^9^, indicating a need for improved and earlier management of the symptoms with concomitant medications, patient education and/or dose modifications to achieve a tolerable dose and treatment persistence.

These exploratory analyses have limitations. First, it should be noted that dose modifications in clinical trials are made in a controlled manner per protocol and in accordance with recommendations to manage hematological and non-hematological toxicities. It is also important to recognize that the monarchE trial was not designed to investigate the impact of dose reductions on efficacy. Following randomization, patients assigned to the treatment arm started abemaciclib at 150 mg, with dose reductions implemented as a measure to manage toxicity for patients who could not tolerate the full dose. Therefore, it was not possible to directly compare different dosing strategies. As a result, the large variability in the number of dose reductions, timing and the treatment duration at each dose level necessitated more sophisticated statistical techniques and several sensitivity analyses that adjusted for confounding factors. Conversely, the process of exploring different analytical approaches also constitutes a strength of these exploratory analyses as they all led to consistent findings, thereby providing confidence in the robustness of the results.

In summary, patients receiving adjuvant abemaciclib should be carefully monitored for possible side effects during treatment, especially if they have features that are associated with higher risk of treatment discontinuation such as age ≥65 years old or ≥4 co-morbidities. Importantly, abemaciclib dose modifications effectively managed side effects and retained more patients on treatment, including those at higher risk for treatment discontinuation. Based on the multiple analyses presented, the efficacy of adjuvant abemaciclib in monarchE was not compromised by dose reductions. Therefore, when required, dose modifications improve tolerability and support the goal of maximizing adherence to maintain the benefit from adjuvant abemaciclib in combination with endocrine therapy for high-risk HR+, HER2- EBC.

## Methods

### Study design and patients

The study design, treatments, and procedures for the monarchE trial (ClinicalTrials.gov identifier: NCT03155997) have previously been published in detail^1,2,9,13^. Patients enrolled to one of two cohorts based on high-risk clinicopathological features. Cohort 1 included patients with ≥4 positive pathologic axillary lymph nodes (ALNs) or 1-3 positive ALNs plus ≥1 of the following: tumor size ≥5 cm or tumor grade 3. Cohort 2 included patients with 1-3 positive ALNs, tumor size <5 cm, tumor grade <3, and centrally assessed Ki-67 ≥ 20%.

Patients were randomized 1:1 to receive standard-of-care adjuvant ET for 5-10 years (physician’s choice) alone or in combination with abemaciclib (150 mg BID) orally for 2 years (on-study treatment period). Abemaciclib dose suspensions and/or up to two 50 mg dose reductions were allowed to manage hematological and non-hematological (diarrhea, increased alanine aminotransferase and/or aspartate aminotransferase, and interstitial lung disease) toxicities, based on toxicity type, severity, persistence, and recurrence. These recommendations have been published previously^9^ and are referenced in Supplementary Table 3.

The monarchE trial was conducted in accordance with the Declaration of Helsinki (1964) and its amendments, the Council for International Organizations of Medical Sciences International Ethical Guidelines, International Conference on Harmonization Good Clinical Practice Guidelines, and all applicable local laws and regulations. The study protocol^14^ was approved by institutional review boards at each site. All patients provided written, informed consent.

### Statistical analyses

Details of the statistical analyses for the monarchE study have been published previously^1,2,9,13^. Data in the current analyses are from a prespecified overall survival interim analysis (data cutoff date: July 1, 2022; median follow-up 42 months)^2^. All patients were off abemaciclib treatment. Patients treated with at least one dose of abemaciclib were included in the analyses.

Among abemaciclib-treated patients, disease characteristics and patient demographics were summarized by the number of dose reductions (0, 1, or 2 dose reductions).

To assess the associations between abemaciclib exposure and the number of dose reductions, treatment duration, cumulative dose, and relative dose intensity (RDI) were summarized by the number of dose reductions. RDI was defined as the average daily dose over the treatment duration each patient received, relative to the full dose.

As dose reductions were expected to be associated with lower RDI, efficacy assessment by RDI-defined patient subgroups was performed as an indirect evaluation of dose reduction impact on efficacy. Patients were divided into three equal-sized subgroups according to their abemaciclib RDI (≤66%, 66–93%, and ≥93%). IDFS, as defined by the STEEP (Standardized Definitions for Efficacy End Points) criteria^15^, was estimated within each subgroup using the Kaplan-Meier method^16^.

To formally assess the impact of dose reductions on IDFS and DRFS, a time-dependent Cox proportional hazards model^17^ was fitted to account for the start and end time of each dose level received. The model assumed that the effect beyond the 2-year abemaciclib treatment period was the same as the last abemaciclib dose received by the patient. Hazard ratios comparing the effect of staying at the full dose versus a reduced dose to 100 or 50 mg were generated with 95% confidence intervals (CI). Additionally, an adjusted model was applied using inverse probability weighting to account for potentially confounding baseline factors that were individually associated with the risk of recurrence, including age, stratification factors, key disease characteristics, and pre-existing co-morbidities.

As a sensitivity analysis to test the assumption on the effect beyond abemaciclib treatment, any IDFS events that occurred beyond completing the 2-year treatment period were censored at the time of discontinuation in a time-dependent Cox proportional hazards model. As treatment discontinuations did not occur randomly, inverse probability censoring weighting was conducted to account for informative censoring wherein hazard ratios were adjusted by potentially confounding baseline factors as well as by factors selected as independently associated with the time to abemaciclib discontinuation due to reasons other than recurrence (TTD). To identify factors independently associated with an increased risk of early abemaciclib discontinuations, patient and disease characteristics were fitted in a multivariate Cox proportional hazards model for time to abemaciclib discontinuations due to reasons other than recurrence, using a stepwise variable selection with entry and retaining a *P*-value threshold of 0.05. The discontinuation rates at 3, 6, 12 and 24 months were estimated using the Kaplan-Meier method^16^ within each subgroup of the selected factors that were independently prognostic of treatment discontinuation.

### Reporting summary

Further information on research design is available in the Nature Research Reporting Summary linked to this article.

### Supplementary information

Updated Supplementary information - Impact of dose reductions on the efficacy of adjuvant abemaciclib for high-risk EBC: analyses from the monarchE study

Reporting Summary

## Data Availability

The datasets generated and/or analyzed during the current study are not publicly available in order to protect patient privacy but are available from the corresponding author on reasonable request.

## Code availability

Data analyses were performed using SAS (enterprise guide 7.1) and R studio server (R version 4.1.2). The code cannot be accessed but may be available from the corresponding author on reasonable request.

## References

[1] Johnston SRD (2020). Abemaciclib combined with endocrine therapy for the adjuvant treatment of HR+, HER2-, node-positive, high-risk, early breast cancer (monarchE). J. Clin. Oncol..

[2] Johnston SRD (2023). Abemaciclib plus endocrine therapy for hormone receptor-positive, HER2-negative, node-positive, high-risk early breast cancer (monarchE): results from a preplanned interim analysis of a randomised, open-label, phase 3 trial. Lancet Oncol.

[3] European Medicines Agency. VERZENIOS™ (abemaciclib). https://www.ema.europa.eu/en/medicines/human/EPAR/verzenios (2022).

[4] Food and Drug Administration (FDA). FDA D.I.S.C.O. Burst Edition: FDA approval of Verzenio (abemaciclib) with endocrine therapy for patients with HR-positive, HER2-negative, node-positive, early breast cancer. https://www.fda.gov/drugs/resources-information-approved-drugs/fda-disco-burst-edition-fda-approval-verzenio-abemaciclib-endocrine-therapy-patients-hr-positive (2023).

[5] Pharmaceuticals and Medical Devices Agency (PMDA). VERZENIO™ (abemaciclib). https://www.pmda.go.jp/drugs/2018/P20181004001/530471000_23000AMX00808_A100_1.pdf (2021).

[6] National Comprehensive Cancer Network. Clinical practice guidelines in oncology. Breast cancer. Version 4. https://www.nccn.org/professionals/physician_gls/pdf/breast.pdf (2023). 10.6004/jnccn.2023.0050 37856213

[7] European Society for Medical Oncology (ESMO). ESMO-MCBS scorecards: abemaciclib. https://www.esmo.org/guidelines/esmo-mcbs/esmo-mcbs-for-solid-tumours/esmo-mcbs-scorecards/scorecard-371-1 (2023).

[8] Rugo HS (2021). Management of abemaciclib-associated adverse events in patients with hormone receptor-positive, human epidermal growth factor receptor 2-negative advanced breast cancer: safety analysis of MONARCH 2 and MONARCH 3. Oncologist.

[9] Rugo HS (2022). Adjuvant abemaciclib combined with endocrine therapy for high-risk early breast cancer: safety and patient-reported outcomes from the monarchE study. Ann. Oncol..

[10] Hadji P (2013). The Patient’s Anastrozole Compliance to Therapy (PACT) Program: a randomized, in-practice study on the impact of a standardized information program on persistence and compliance to adjuvant endocrine therapy in postmenopausal women with early breast cancer. Ann. Oncol..

[11] Ziller V (2009). Adherence to adjuvant endocrine therapy in postmenopausal women with breast cancer. Ann. Oncol..

[12] Hershman DL (2011). Early discontinuation and non-adherence to adjuvant hormonal therapy are associated with increased mortality in women with breast cancer. Breast Cancer Res. Treat..

[13] Harbeck N (2021). Adjuvant abemaciclib combined with endocrine therapy for high-risk early breast cancer: updated efficacy and Ki-67 analysis from the monarchE study. Ann. Oncol..

[14] Eli Lilly and Company. monarchE Protocol I3Y-MC-JPCF(e). https://classic.clinicaltrials.gov/ProvidedDocs/97/NCT03155997/Prot_000.pdf (2019).

[15] Hudis CA (2007). Proposal for standardized definitions for efficacy end points in adjuvant breast cancer trials: the STEEP system. J. Clin. Oncol..

[16] Kaplan EL, Meier P (1958). Nonparametric estimation of incomplete observation. J. Am. Stat. Assoc..

[17] Therneau T. M. & Grambsch P. M. Modeling Survival Data: Extending the Cox Model. 10.1007/978-1-4757-3294-8 (Springer, 2000).

