# Supplementary material for: Impact of dose reductions on adjuvant abemaciclib efficacy for patients with high-risk early breast cancer: analyses from the monarchE study
Source: NPJ Breast Cancer. 2024 Apr 26;10:34. doi: 10.1038/s41523-024-00639-1 (PMC11053007; doi:10.1038/s41523-024-00639-1)
Supplement: Supplementary file 1 — Updated Supplementary information - Impact of dose reductions on the efficacy of adjuvant abemaciclib for high-risk EBC: analyses from the monarchE study [file 41523_2024_639_MOESM1_ESM.pdf]

Supplementary Information - Impact of dose reductions on adjuvant abemaciclib efficacy for patients with high-risk early breast cancer: analyses from the monarchE study

Supplementary Table 1. Yearly invasive disease-free survival rates by abemaciclib relative dose intensity subgroup

**Analysis**		**IDFS rates, % (95% CI)[b]**		
**Population**	**RDI group[a]**	**2 years**	**3 years**	**4 years**
Intent-to-treat	≤66%	94.1 (92.3, 95.5)	91.2 (89.1, 92.9)	87.1 (84.0, 89.7)
	66%-93%	92.5 (90.6, 94.1)	89.5 (87.2, 91.3)	86.4 (83.6, 88.7)
	≥93%	91.5 (89.4, 93.1)	86.8 (84.4, 88.9)	83.7 (80.7, 86.3)
Cohort 1[c]	≤66%	94.1 (92.3, 95.6)	91.0 (88.8, 92.8)	87.2 (84.0, 89.8)
	66%-93%	92.2 (90.2, 93.9)	89.0 (86.6, 90.9)	86.1 (83.3, 88.5)
	≥93%	91.4 (89.2, 93.1)	86.3 (83.7, 88.5)	83.1 (79.9, 85.8)

Data cutoff date: July 01, 2022.

[a]RDI was defined as the average daily dose of abemaciclib received by each patient over the treatment duration, relative to the full dose (150 mg twice per day). For efficacy analyses, patients were divided into three equal-sized subgroups according to their abemaciclib RDI.

[b]Estimated by the Kaplan-Meier method.

[c]Cohort 1 included patients with ≥4 positive pathologic ALNs or 1-3 positive ALNs plus tumor size ≥5 cm and/or tumor grade 3.

ALN, axillary lymph nodes; CI, confidence interval; IDFS, invasive disease-free survival; RDI, relative dose intensity.

Supplementary Table 2. Discontinuation rates in the abemaciclib arm of monarchE by baseline patient characteristics

			Rates of Discontinuation, % (95% CI) [a]		
**Factors**		***N***	**6 months**	**12 months**	**24 months**
Geographic region	North America/Europe	1458	16.5 (14.7, 18.5)	23.5 (21.3, 25.7)	32.2 (29.8, 34.7)
	Asia	573	10.3 (8.0, 13.0)	13.9 (11.2, 16.9)	18.5 (15.4, 21.8)
	Other	760	12.1 (9.8, 14.5)	15.8 (13.3, 18.5)	21.2 (18.2, 24.4)
Menopausal status	Premenopausal	1217	9.3 (7.8, 11.0)	12.8 (10.9, 14.7)	18.2 (16.0, 20.5)
	Postmenopausal	1574	17.7 (15.8, 19.6)	24.6 (22.5, 26.8)	32.9 (30.4, 35.3)
Age group	<65 years	2361	11.6 (10.3, 12.9)	16.3 (14.9, 17.9)	22.7 (21.0, 24.5)
	≥65 years	430	27.6 (23.4, 31.9)	36.4 (31.8, 41.0)	46.6 (41.5, 51.6)
Baseline ECOG PS	0	2392	13.7 (12.4, 15.2)	18.7 (17.1, 20.3)	25.2 (23.4, 27.0)
	1	399	15.8 (12.4, 19.6)	24.0 (19.9, 28.3)	33.9 (29.2, 38.7)
Number of positive nodes	1–3	1115	16.1 (14.0, 18.3)	22.3 (19.8, 24.8)	30.5 (27.8, 33.3)

	4–9	1096	13.2 (11.3, 15.3)	17.9 (15.7, 20.3)	24.5 (21.9, 27.2)
	≥10	573	11.6 (9.1, 14.4)	16.8 (13.8, 20.0)	22.1 (18.5, 25.9)
Number of unique pre-existing comorbidities	0	460	9.4 (6.9, 12.3)	11.8 (9.1, 15.0)	16.2 (13.0, 19.8)
	1–3	1373	12.9 (11.2, 14.7)	17.7 (15.7, 19.8)	23.7 (21.5, 26.1)
	≥4	958	17.9 (15.6, 20.4)	25.5 (22.8, 28.3)	35.1 (32.0, 38.3)

Data cutoff date: July 01, 2022.

CI, confidence interval; ECOG PS, Eastern Cooperative Oncology Group performance status;
[a]Discontinuation rates (95% CI) during the on-study treatment period were estimated using the Kaplan-Meier method.

Supplementary Table 3. Recommendation for management of adverse events.

Adverse Events		CTCAE Grade	Dose Modifications
Hematologic Toxicities[a]	Monitor complete blood counts prior to the start of abemaciclib therapy, every 2 weeks for the first 2 months, monthly for the next 2 months, and as clinically indicated.	Grade 1 or 2	No dose modification is required.
		Grade 3	Suspend dose until toxicity resolves to ≤Grade 2. Dose reduction is not required.
		Grade 3 recurrent, or Grade 4	Suspend dose until toxicity resolves to ≤Grade 2. Resume at next lower dose.
Diarrhoea	At the first sign of loose stools, start treatment with antidiarrheal agents and increase intake of oral fluids	Grade 1	No dose modification is required.
		Grade 2	If toxicity does not resolve within 24 hours to ≤Grade 1, suspend dose until resolution. No dose reduction is required.
		Grade 2 that persists or recurs after resuming the same dose despite maximal supportive measures	Suspend dose until toxicity resolves to ≤Grade 1. Resume at next lower dose
		Grade 3 or 4 or requires hospitalization	Suspend dose until toxicity resolves to ≤Grade 1. Resume at next lower dose.
Hepatotoxicity	Monitor ALT, AST, and serum bilirubin prior to the start of abemaciclib therapy, every 2 weeks for the first 2 months, monthly for the next 2 months, and as clinically indicated.	Grade 1 (>ULN-3.0 x ULN) Grade 2 (>3.0-5.0 x ULN), WITHOUT increase in total bilirubin above 2 x ULN	No dose modification is required.
		Persistent or Recurrent Grade 2, or Grade 3 (>5.0- 20.0 x ULN), WITHOUT increase in total bilirubin above 2 x ULN	Suspend dose until toxicity resolves to baseline or Grade 1. Resume at next lower dose.
		Elevation in AST and/or ALT >3 x ULN WITH total bilirubin >2 x ULN, in the absence of cholestasis	Discontinue Abemaciclib
		Grade 4 (>20.0 x ULN)	Discontinue Abemaciclib
Interstitial Lung Disease/Pneumonitis		Grade 1 or 2	No dose modification is required
		Persistent or recurrent Grade 2 toxicity that does not resolve with maximal supportive measures within 7 days to baseline or Grade 1	Suspend dose until toxicity resolves to baseline or ≤Grade 1. Resume at next lower dose
		Grade 3 or 4	Discontinue Abemaciclib
Other Toxicities[b]		Grade 1 or 2	No dose modification is required.
		Persistent or recurrent Grade 2 toxicity that does not resolve with maximal supportive measures within 7 days to baseline or Grade 1	Suspend dose until toxicity resolves to baseline or ≤Grade 1. Resume at next lower dose
		Grade 3 or 4	Suspend dose until toxicity resolves to baseline or ≤Grade 1. Resume at next lower dose.

A dose reduction corresponds to a reduction of 50 mg of abemaciclib at a time. Discontinue abemaciclib for patients unable to tolerate 50 mg twice daily. [a]If blood cell growth factors are required, suspend abemaciclib dose for at least 48 hours after the last dose of blood cell growth factor and until toxicity resolves to ≤Grade 2. Resume at next lower dose unless already performed for the toxicity that led to the use of the growth factor. Growth factor use as per current treatment guidelines. [b]Excluding diarrhea, hematologic toxicity, hepatotoxicity,ILD/pneumonitis, and VTEs. Abbreviations: AE, adverse event; ALT, alanine aminotransferase; ILD, interstitial lung disease; ULN, upper limit of normal. Reproduced from Rugo, H. S. et al. *Oncologist* **26,** e53–e65 (2021) and Abemaciclib package insert

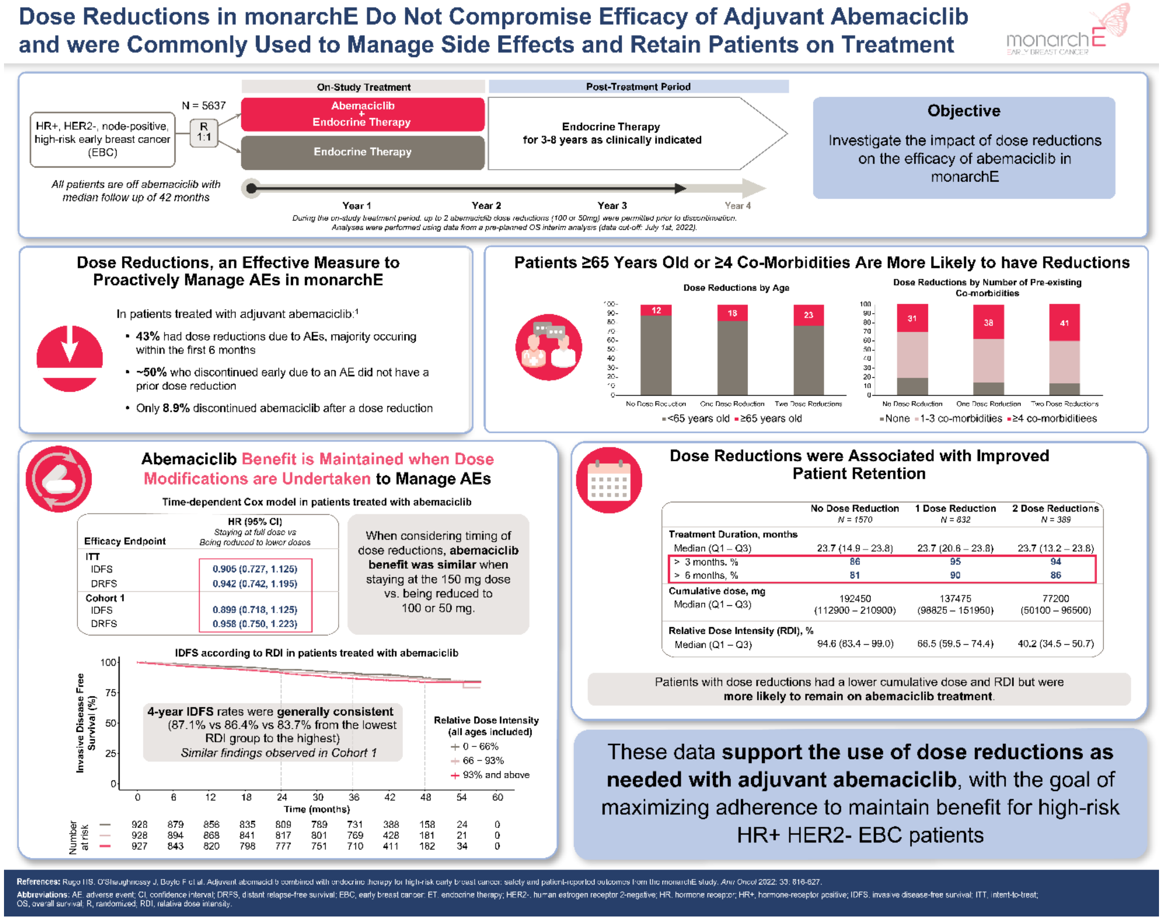

Supplementary Figure 1. Summary

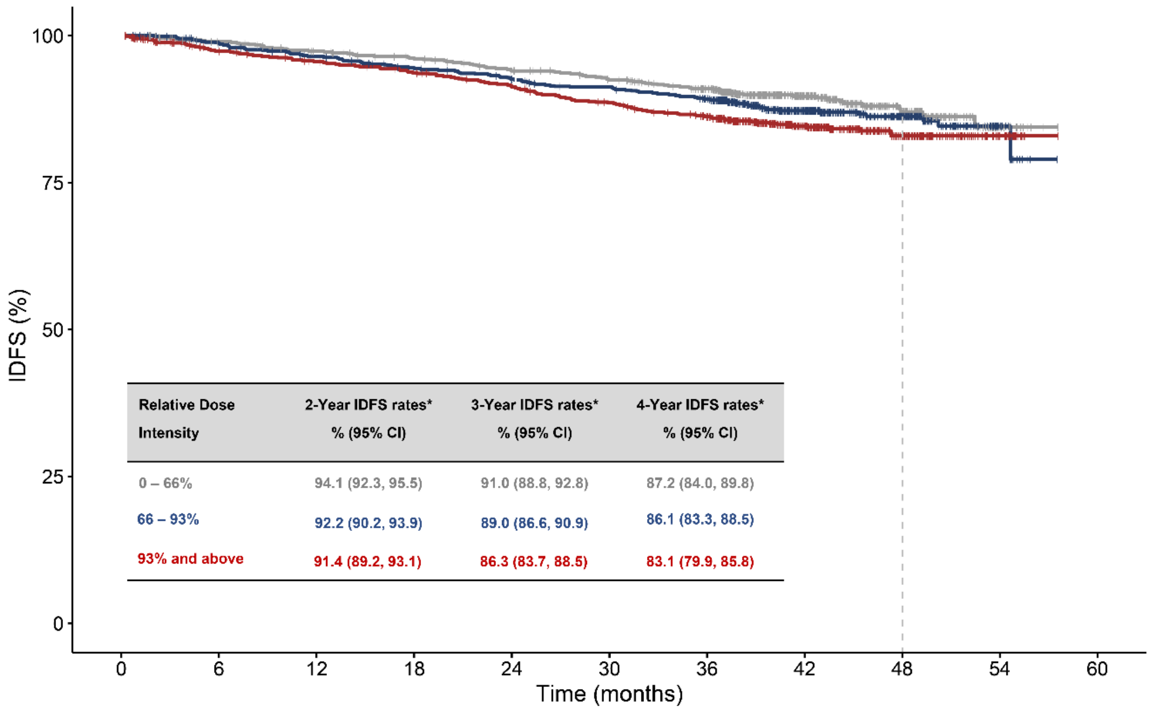

Relative Dose Intensity	2-Year IDFS rates* % (95% CI)	3-Year IDFS rates* % (95% CI)	4-Year IDFS rates* % (95% CI)
0 – 66%	94.1 (92.3, 95.5)	91.0 (88.8, 92.8)	87.2 (84.0, 89.8)
66 – 93%	92.2 (90.2, 93.9)	89.0 (86.6, 90.9)	86.1 (83.3, 88.5)
93% and above	91.4 (89.2, 93.1)	86.3 (83.7, 88.5)	83.1 (79.9, 85.8)

Number at risk

(grey)	829	789	768	749	725	707	663	367	158	24	0
(blue)	855	822	798	772	750	734	709	407	181	21	0
(red)	847	772	752	731	710	684	647	386	181	34	0

Supplementary Figure 2. Invasive disease-free survival by relative dose intensity subgroup in patients treated with abemaciclib (Patients in Cohort 1) RDI was defined as the average daily dose of abemaciclib received by each patient over the treatment duration, relative to the full dose (150 mg twice per day). *Estimated by the Kaplan-Meier method. For efficacy analyses, patients were divided into three equal-sized subgroups according to their abemaciclib RDI. Data cutoff date: July 01, 2022.

CI, confidence interval; IDFS, invasive disease-free survival; RDI, relative dose intensity

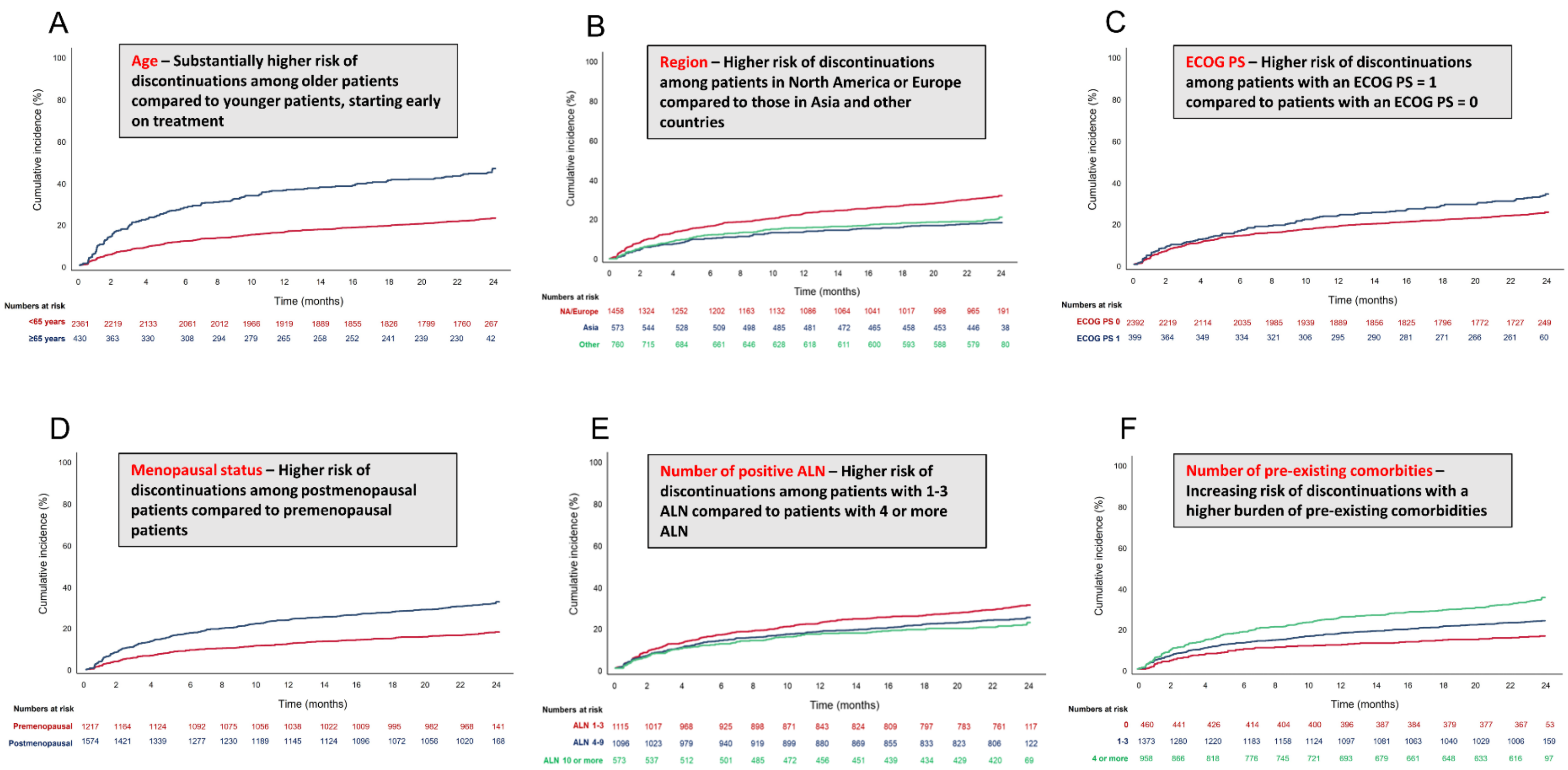

Supplementary Figure 3. Discontinuations in the abemaciclib arm of the monarchE trial. Discontinuations during the on-study treatment period for subgroups categorized by baseline factors, including A) age; B) geographic region; C) ECOG PS; D) menopausal status; E) number of positive ALN; and F) number of pre-existing comorbidities.

ALN, axillary lymph nodes; ECOG PS, Eastern Cooperative Oncology Group performance status; NA, North America
