# Supplementary material for: Impact of dose reductions on adjuvant abemaciclib efficacy for patients with high-risk early breast cancer: analyses from the monarchE study
Source: NPJ Breast Cancer. 2024 Apr 26;10:34. doi: 10.1038/s41523-024-00639-1 (PMC11053007; doi:10.1038/s41523-024-00639-1)
Supplement: Supplementary file 2 — Reporting Summary [file 41523_2024_639_MOESM2_ESM.pdf]

nature portfolio

Corresponding author(s): Matthew Goetz

Last updated by author(s): Feb 8, 2024

Reporting Summary

Nature Portfolio wishes to improve the reproducibility of the work that we publish. This form provides structure for consistency and transparency in reporting. For further information on Nature Portfolio policies, see our Editorial Policies and the Editorial Policy Checklist.

Statistics

For all statistical analyses, confirm that the following items are present in the figure legend, table legend, main text, or Methods section.

n/a	Confirmed	
☐	☒	The exact sample size (*n*) for each experimental group/condition, given as a discrete number and unit of measurement
☐	☒	A statement on whether measurements were taken from distinct samples or whether the same sample was measured repeatedly
☒	☐	The statistical test(s) used AND whether they are one- or two-sided<br>*Only common tests should be described solely by name; describe more complex techniques in the Methods section.*
☐	☒	A description of all covariates tested
☐	☒	A description of any assumptions or corrections, such as tests of normality and adjustment for multiple comparisons
☐	☒	A full description of the statistical parameters including central tendency (e.g. means) or other basic estimates (e.g. regression coefficient) AND variation (e.g. standard deviation) or associated estimates of uncertainty (e.g. confidence intervals)
☒	☐	For null hypothesis testing, the test statistic (e.g. *F*, *t*, *r*) with confidence intervals, effect sizes, degrees of freedom and *P* value noted<br>*Give P values as exact values whenever suitable.*
☒	☐	For Bayesian analysis, information on the choice of priors and Markov chain Monte Carlo settings
☒	☐	For hierarchical and complex designs, identification of the appropriate level for tests and full reporting of outcomes
☐	☒	Estimates of effect sizes (e.g. Cohen's *d*, Pearson's *r*), indicating how they were calculated

Our web collection on statistics for biologists contains articles on many of the points above.

Software and code

Policy information about availability of computer code

Data collection	Refer to study protocol for data collection details.
Data analysis	Data analyses were performed using SAS (enterprise guide 7.1) and R studio server (R version 4.1.2)

For manuscripts utilizing custom algorithms or software that are central to the research but not yet described in published literature, software must be made available to editors and reviewers. We strongly encourage code deposition in a community repository (e.g. GitHub). See the Nature Portfolio guidelines for submitting code & software for further information.

Data

Policy information about availability of data

All manuscripts must include a data availability statement. This statement should provide the following information, where applicable:

- Accession codes, unique identifiers, or web links for publicly available datasets
- A description of any restrictions on data availability
- For clinical datasets or third party data, please ensure that the statement adheres to our policy

The datasets generated and/or analyzed during the current study are not publicly available in order to protect patient privacy but are available from the corresponding author on reasonable request.

Research involving human participants, their data, or biological material

Policy information about studies with human participants or human data. See also policy information about sex, gender (identity/presentation), and sexual orientation and race, ethnicity and racism.

Reporting on sex and gender	Female (any menopausal status) and male patients over 18 years of age with HR+ and HER2- disease were eligible. The population was predominantly female (99.4%) and postmenopausal (56.5%).
Reporting on race, ethnicity, or other socially relevant groupings	Patients from 603 sites in 38 countries were enrolled primarily from North America/Europe in both treatment groups (~52%). 20% of patients were from Asia.
Population characteristics	From July 2017 to August 2019, 5,637 patients from 603 sites in 38 countries were randomly assigned 1:1 to receive either abemaciclib plus ET or ET alone. The population had a median age of 51.0 years (12.6% patients < 40 years) and was predominantly female (99.4%), and postmenopausal (56.5%) at the time of diagnosis. Nearly 60% of patients were eligible on the basis of four or more nodes.
Recruitment	From July 2017 to August 2019, 5,637 patients from 603 sites in 38 countries were randomly assigned 1:1 to receive either abemaciclib plus ET or ET alone.
Ethics oversight	The study protocol and all amendments received approval from ethical/institutional review boards before implementation, and all patients gave written informed consent. Executive and Global Steering Committees provided oversight of the conduct of the trial, and an independent data monitoring committee reviewed the safety data approximately every 6 months and efficacy data at prespecified interim analyses

Note that full information on the approval of the study protocol must also be provided in the manuscript.

Field-specific reporting

Please select the one below that is the best fit for your research. If you are not sure, read the appropriate sections before making your selection.

☒ Life sciences ☐ Behavioural & social sciences ☐ Ecological, evolutionary & environmental sciences

For a reference copy of the document with all sections, see nature.com/documents/nr-reporting-summary-flat.pdf

Life sciences study design

All studies must disclose on these points even when the disclosure is negative.

Sample size	There were 5,637 patients from 603 sites in 38 countries were randomly assigned 1:1 to receive either abemaciclib plus ET or ET alone. The study was powered at approximately 85% to detect the superiority of abemaciclib plus endocrine therapy versus endocrine therapy alone in invasive disease-free survival, with an assumed HR of 0·73 and two-sided alpha level of 0·05. This required approximately 390 invasive disease-free survival events in the ITT population
Data exclusions	Since this manuscript was intended to assess the impact of abemaciclib dose reductions on efficacy, the analyses included patients who received at least one dose of abemaciclib in the study. Patients who only received ET alone were excluded from the analyses.
Replication	For all the exploratory analyses reported in the MS, independent validation was taken to verify the replicability of the analysis results.
Randomization	An interactive Web response system was used to randomly assign patients (1:1) to receive either abemaciclib (150 mg twice daily on a continuous dosing schedule) plus ET or ET alone. Stratification factors included previous chemotherapy (neoadjuvant, adjuvant, or none), menopausal status (as determined at the time of breast cancer diagnosis), and region (North America/Europe, Asia, or other).
Blinding	Although this was an open-label study, the sponsor and all investigative sites remained blinded to treatment group assignments for aggregate data until the study was confirmed as positive.

Reporting for specific materials, systems and methods

We require information from authors about some types of materials, experimental systems and methods used in many studies. Here, indicate whether each material, system or method listed is relevant to your study. If you are not sure if a list item applies to your research, read the appropriate section before selecting a response.

Materials & experimental systems

n/a	Involved in the study
☒	☐ Antibodies
☒	☐ Eukaryotic cell lines
☒	☐ Palaeontology and archaeology
☒	☐ Animals and other organisms
☐	☒ Clinical data
☒	☐ Dual use research of concern
☒	☐ Plants

Methods

n/a	Involved in the study
☒	☐ ChIP-seq
☒	☐ Flow cytometry
☒	☐ MRI-based neuroimaging

Clinical data

Policy information about clinical studies

All manuscripts should comply with the ICMJE guidelines for publication of clinical research and a completed CONSORT checklist must be included with all submissions.

Clinical trial registration: NCT03155997

Study protocol: https://classic.clinicaltrials.gov/ProvidedDocs/97/NCT03155997/Prot_000.pdf

Data collection: Patients from 603 sites in 38 countries were enrolled. Patient recruitment from 2017-2019

Outcomes: The primary endpoint was invasive disease-free survival, defined as time from randomization to the first occurrence of local or regional recurrence, contralateral recurrence, second primary non-breast invasive cancer, distant recurrence, or death attributable to any cause according to the STEEP criteria. Secondary endpoints were invasive disease-free survival in patients with high Ki-67 index (≥20%), distant relapse-free survival (defined as time from randomisation to the first occurrence of distant recurrence or death attributable to any cause), overall survival (defined as time from randomisation to the date of death due to any cause), patient-reported outcomes, and safety.

Plants

Seed stocks: N/A

Novel plant genotypes: N/A

Authentication: N/A
